# Anti-Müllerian Hormone and OPU-ICSI Outcome in the Mare

**DOI:** 10.3390/ani11072004

**Published:** 2021-07-05

**Authors:** Marion Papas, Jan Govaere, Sofie Peere, Ilse Gerits, Margot Van de Velde, Daniel Angel-Velez, Tine De Coster, Ann Van Soom, Katrien Smits

**Affiliations:** 1 Department of Reproduction, Obstetrics and Herd Health, Faculty of Veterinary Medicine, Ghent University, Salisburylaan 133, 9820 Merelbeke, Belgium; jan.govaere@ugent.be (J.G.); sofie.peere@ugent.be (S.P.); ilse.gerits@ugent.be (I.G.); margot@praktivet.be (M.V.d.V.); daniel.angelvelez@ugent.be (D.A.-V.); tine.decoster@ugent.be (T.D.C.); ann.vansoom@ugent.be (A.V.S.); katrien.smits@ugent.be (K.S.); 2 Research Group in Animal Sciences-INCA-CES, Universidad CES, 050021 Medellin, Colombia

**Keywords:** anti-müllerian hormone, mare, OPU, ICSI

## Abstract

**Simple Summary:**

The in vitro production of equine embryos, using ovum pick-up (OPU) and intracytoplasmic sperm injection (ICSI), is gaining popularity for breeding sport horses. However, the results of this complicated procedure are variable and hard to predict. Therefore, the aim of this study was to evaluate anti-Müllerian hormone (AMH), a factor which has been linked to reproductive success in human and cattle, as an indicator of the OPU-ICSI outcome in horses. Therefore, for 103 mares subjected to commercial OPU-ICSI, the AMH level was measured in the blood at the moment of OPU and linked to the number of oocytes and embryos produced by ICSI. We found that mares with a high level of AMH gave rise to a better oocyte collection and a higher number of embryos. However, since mares with a low AMH value could also produce embryos, a single measurement of the AMH in the blood is not sufficient as an independent predictor of the OPU-ICSI outcome in the horse.

**Abstract:**

Anti-Müllerian hormone (AMH) reflects the population of growing follicles and has been related to mammalian fertility. In the horse, clinical application of ovum pick-up and intracytoplasmic sperm injection (OPU-ICSI) is increasing, but results depend largely on the individuality of the mare. The aim of this study was to assess AMH as a predictor for the OPU-ICSI outcome in horses. Therefore, 103 mares with a total follicle count above 10 were included in a commercial OPU-ICSI session and serum AMH was determined using ELISA. Overall, the AMH level was significantly correlated with the number of aspirated follicles and the number of recovered oocytes (*p* < 0.001). Mares with a high AMH level (≥2.5 µg/L) yielded significantly greater numbers of follicles (22.9 ± 1.2), oocytes (13.5 ± 0.8), and blastocysts (2.1 ± 0.4) per OPU-ICSI session compared to mares with medium (1.5–2.5 µg/L) or low AMH levels (<1.5 µg/L), but no significant differences in blastocyst rates were observed. Yet, AMH levels were variable and 58% of the mares with low AMH also produced an embryo. In conclusion, measurement of serum AMH can be used to identify mares with higher chances of producing multiple in vitro embryos, but not as an independent predictor of successful OPU-ICSI in horses.

## 1. Introduction

During the last decade, considerable progress has been made in the production of embryos by combining ovum pick-up (OPU) with oocyte in vitro maturation (IVM) and subsequent intracytoplasmic sperm injection (ICSI) in sport horses [1,2,3]. This methodology provides a solution for subfertility in both mares and stallions [1,4] and might lead to a more efficient and economically profitable use of fertile mares. The advantages include the necessity for only a small amount of scarce and expensive semen [2,5], the production of embryos at any moment of the year, without compromising sport engagements, and the possibility to determine the gender of the embryo before cryopreservation and transfer [1]. Individuality of the mare and the number of retrieved oocytes come across as the two main parameters influencing a successful outcome of OPU-ICSI [5,6], though male factors have to be considered too [7].

Anti-Müllerian hormone (AMH), also known as Müllerian inhibiting substance (MIS), is a dimeric glycoprotein identified as a member of the transforming growth factor β superfamily of growth and differentiation [8]. Anti-Müllerian hormone is exclusively expressed by male and female gonadal somatic cells in mammals [8,9]. Its fundamental function is to inhibit the development of the paramesonephric ducts (Müllerian ducts) during fetal sex differentiation in the male [10]. In females, expression of AMH is detected after birth, in the granulosa cells of early primary, preantral and small antral follicles [9]. In the postnatal ovary, AMH appears to play an important role in the recruitment of primordial follicles by preventing them from entering the pool of growing follicles prior to selection, and consequently avoiding the premature depletion of the ovarian reserve [11]. Furthermore, AMH also modulates follicular development by decreasing the sensitivity of the pre-antral follicle to FSH [12].

A single measurement of the AMH level in the blood appears to be a reliable marker of the reproductive capacity in women [13] and domestic animals [14]. Indeed, since AMH is secreted into the circulation by the ovary, serum concentration of this hormone is used to assess the ovarian reserve among species [15] and highly reflects the pre-antral and early antral follicle population in the ovaries of women [16,17], mice [18], cattle [19], bitches [20], and mares [21,22]. The relationship between AMH concentration and follicle population is species-specific. Circulating AMH levels are strongly linked to the number of 2–6 mm diameter follicles in women [23], 3–7 mm in cattle [24], and 1–5 mm in goats [25]. In mares, follicles sized between 6 and 20 mm diameter appear to be most reflective of serum AMH concentrations [26].

In women, AMH measurements are used for many clinical applications, such as assessment of ovarian damage after therapy or estimation of the ovarian reserve in women suffering from polycystic ovary syndrome (PCOS) [27]. Moreover, the AMH concentration appears to be a good predictor of the oocyte quality [28] and has been positively associated with outcomes of in vitro fertilization, including live birth rates [29,30]. In cattle and goats, measurement of the circulating AMH concentration provides practical information to detect donors with the capacity to produce low or high numbers of in vivo embryos after super-ovulatory treatment [25,31,32]. Similar results have been observed for production of in vitro embryos in Holstein cows [31]. In swine, a single measurement of serum AMH is positively linked to gonadotropin responsiveness and subsequent fertility [33,34].

In horses, the AMH concentration is used as an endocrine marker of reproductive disorders in both mare and stallion [35,36]. In cryptorchid stallions, determination of serum AMH levels increases the diagnostic accuracy when testosterone concentrations are inconclusive [37]. In mares, measurement of AMH to diagnose a granulosa theca cell tumor appears to be more specific than inhibin, testosterone, and progesterone concentrations [35,38]. Assessment of AMH levels in prepubertal mares has also been shown to predict the ovarian reserve after puberty [39]. Likewise, circulating AMH represents a good endocrinological marker to assess ovarian function following immunocontraception in mares with the porcine zona pellucida vaccines (pZP) [40]. Recently, Ball et al. [41] observed a positive correlation between serum AMH concentration and pregnancy rates after natural mating.

Finally, serum AMH levels seem to be repeatable within individual normal cyclic mares and are not affected by the reproductive stage [35,41]. Although it is worth mentioning that wide variations of serum AMH levels are observed between mares [35,42], the repeatability of the circulating AMH concentration makes this hormone a candidate as a prospective diagnostic tool for the outcome of commercial OPU-ICSI sessions.

Against this background, and in order to better advise customers in their expectations, a retrospective study was designed to determine whether a single measurement of serum AMH at the time of OPU could be a useful tool to predict the OPU-ICSI outcome in mares.

## 2. Materials and Methods

### 2.1. Animals

A total of 103 different warmblood mares between 2 and 24 years old who were presented for a commercial OPU-ICSI session at our clinic between September 2019 and March 2021 were included in the study. Out of these 103 mares, 14 were subjected to three repeated OPU-ICSI procedures. Mares were previously monitored by transrectal ultrasonography (7.5-MHz transducter, transrectal linear probe, MyLabOne, Esaote, Genoa, Italy) and only selected for OPU when the total follicle count was above 10, regardless of the stage of the reproductive cycle. All follicles with a diameter between 5 and 25 mm were counted.

Following the OPU procedure, ICSI was performed with frozen-thawed semen of 29 different warmblood stallions (min. 1, max. 26). Sessions using semen subjected to thawing, dilution, and refreezing were not included in the analysis.

### 2.2. Transvaginal Ultrasound-Guided Follicle Aspiration

Peri-operative treatment consisted of administration of benzylpenicillin (Penikel, Kela, Sint Niklaas, Belgium, 20,000 IU/kg) and flunixin meglumin (Wellicox, Ceva Santé Animale, Naaldwijk, The Netherlands, 1.1 mg/kg). During the procedure, mares were under sedation with detomidine hydrochloride (Domidine, Eurovet Animal Health BV, Bladel, The Netherlands, 0.01 mg/kg) and butorphanol tartrate (Dolorex, MSD Animal Health, Sint-Lambrechts-Woluwe, Belgium, 0.01 mg/kg). N-butylscopolammonium bromide (Buscopan, Boehringer Ingelheim, Brussel, Belgium, 0.3 mg/kg) was administered to prevent bowel contractions. The urine bladder was not routinely cannulated during the procedure. After aseptic preparation of the perineal zone, the transvaginal probe was inserted into the vagina. The ovary was then manipulated and fixed by rectal palpation against the vaginal wall. A 12-G double-lumen needle, connected to a prewarmed collection bottle, was guided by a channel of the transvaginal probe into the antral follicles. All visible follicles were punctured, aspirated, and flushed eight times with 1 to 3 mL of prewarmed flushing medium (Equiplus, Minitube, Tiefenbach, Germany), depending on the follicle size. When the follicles were collapsing, the follicle wall was scraped by rotating the needle. Once all the follicles from one ovary were aspirated, the collection bottle was brought directly to the laboratory.

Clinical follow-up was ensured by ultrasound if considered necessary. All mares underwent the OPU procedures well without any complications.

### 2.3. In Vitro Maturation and ICSI

Follicular fluid was poured through a sterile 70 µm oocyte filter (Cell strainer, BD Biosciences, Falcon^®^, Erembodegem, Belgium) and cumulus-oocyte-complexes (COCs) were collected in Medium 199 with Hank’s salts (Gibco, Life Technologies, Merelbeke, Belgium). For 47 mares, oocytes were kept overnight in a commercially available holding medium (Emcare, Agtech, Zulte, Belgium (*n* = 38) or Syngro, Vetoquinol, Belgium (*n* = 9)) at room temperature (22 °C) prior to in vitro maturation (IVM) in order to organize subsequent ICSI. For the other 56 mares, oocytes were transferred directly to maturation medium (Medium 199 with Earl’s salts (Gibco) containing 10% (*v*/*v*) FBS (Gibco), 9.4 μg/mL follicle stimulating hormone, and 1.88 μg/mL luteinising hormone (Stimufol, Reprobiol, Ouffet, Belgium)). Maturation was performed in groups of 2–21 COCs in 100–500 µL maturation medium under oil (CooperSurgical, Venlo, The Netherlands) at 38.5 °C in 5% CO_2_ in air for 30 h on average (min. 26.5 h; max 32.3 h). Semen was prepared by thawing a small piece of a straw in 1 mL of preheated (38.5 °C) G-MOPS (Vitrolife, Londerzeel, Belgium), followed by washing in 5 mL of G-MOPS. After centrifugation for 10 min at 400× *g* at 26 °C, the supernatants were removed and the sperm pellet was resolved in 200 µL of G-MOPS. Immediately before ICSI, a small volume of the sperm suspension was added to a 5 μL droplet of 7.5% polyvinylpyrrolidone (CooperSurgical, Venlo, The Netherlands). Oocytes were denuded by pipetting in 0.1% hyaluronidase (Sigma-Aldrich, Bornen, Belgium) in Medium 199 with Hank’s salts with 10% FBS. Intracytoplasmic sperm injection was performed as described previously [43] with slight modifications. Mature oocytes (MII), indicated by an extruded polar body, were injected by piezo drill assisted intracytoplasmic sperm injection (PrimeTech, Nakamukaihara Tsuchiura Ibaraki, Japan; speed 3–4, intensity 6–8). Presumed zygotes were cultured in groups of 1 to 16 in 20 µL droplets of DMEM/F-12 (Gibco) with 10% (*v*/*v*) FBS under oil at 38.2 °C in a humidified atmosphere of 5% O_2_, 5% CO_2_, and 90% N_2_. Cleavage rate was evaluated 2 or 3 days after ICSI and blastocyst development was monitored daily from day six onwards until day 13.

### 2.4. AMH Assay

Blood samples were obtained by jugular venipuncture at the start of the OPU procedure using standard sampling tubes without separating gel. Sera were directly sent to an external specialized laboratory (Algemeen Medisch Laboratorium (AML), Sonic Healthcare Benelux, Antwerp, Belgium) and samples were maintained at 2–8 °C until analysis. Anti-Müllerian hormone concentrations were determined using a commercial kit Elecsys AMH Plus (Roche Diagnostics, Risch-Rotkreuz, Switzerland), a one-step sandwich method based upon electrochemiluminescence immunoassay for the in vitro quantitative determination of AMH. The electrochemiluminescence immunoassay was performed with cobas e411 (Roche Diagnostics, Risch-Rotkreuz, Switzerland) immunoassay analyzer. The analyses were performed no more than 24 h after collection and according to the manufacturer’s instructions. Briefly, 50 µL of the tested sample was incubated for nine minutes with a biotinylated monoclonal mammalian AMH-specific antibody and a monoclonal mammalian AMH-specific antibody labeled with a ruthenium complex (Tris (2,2-bipytidyl)ruthenium(II)-complex[Ru(bpy)3]^2+^) to form a sandwich complex. The complex was then incubated with streptavidin microplarticles for another nine minutes. The microparticles of the reaction mixture were then magnetically captured onto the surface of an electrode and unbound substances were removed. Removal of the unbound substances was performed by washing the streptavidin-coated microparticles with the solution ProCell, Elecsys, cobas (Roche Diagnostics, Risch-Rotkreuz, Switzerland). Chemiluminescent emission was induced through application of a voltage to the electrode and then measured by a photomultiplier. Results were determined via a calibration curve generated by 2-point calibration and a master curve provided by the manufacturer. The analyzer provides automatically the AMH concentration of each sample. Controls are realized for each analysis. Kit controls were provided by the company and the lower and upper limits of each control were determined by the company and were read into the device via the control barcode. The intra- and inter-assay precision of the kit were ≤1.3% and ≤4.1%, respectively. The limits of blank, quantitation and detection were, respectively 0.007 ng/mL, 0.030 µg/L and 0.010 µg/L. The method was standardized against the Beckman Coulter AMH Gen II ELISA assay.

### 2.5. Statistical Analysis

For the general analysis, data from 103 OPU-ICSI sessions of different warmblood mares were analyzed. The relation between OPU-ICSI outcome, including numbers of follicles aspirated, numbers of oocytes recovered, and numbers of blastocysts produced, and serum AMH concentration was examined by linear regression. Association of the AMH concentration with the number of follicles aspirated and number of oocytes recovered was evaluated using Spearman’s correlation coefficient by rank. For the categorical variables, a chi-squared test was carried out. Comparisons among means were performed using the Bonferroni adjustment. Effect of individual stallions on the blastocyst rate and in the number of blastocysts produced per OPU-ICSI session was determined using the Kruskal–Wallis test. For the analysis of the variation within individual mares, data from 42 OPU-ICSI sessions were included (three OPU-ICSI sessions of 14 mares). Repeated-measures ANOVA was used to analyze changes in AMH average concentration with OPU session. The nonparametric test of Friedman was performed to detect differences in blastocyst rate across the OPU-ICSI sessions. Statistical analysis was performed by the Statistical Package for the Social Sciences for Windows (SPSS, Armonk, NY, USA), version 27. Differences with *p* < 0.05 were considered significant. All data are presented as mean ± standard error of the mean (SEM), except for the correlation studies.

## 3. Results

Anti-Müllerian hormone ranged from 0.6 to 4.1 µg/L (2.0 ± 0.1 µg/L) with a median of 1.8 µg/L. Overall, on average of 19.3 (±0.6) follicles were aspirated, 10.7 (±0.4) oocytes were recovered, 7 (±0.3) oocytes were subjected to ICSI, 4.5 (±0.2) injected oocytes were cleaved, and 1.4 (±0.2) blastocysts were produced per OPU session. From the 103 individual OPU-ICSI sessions, 712 mature oocytes were injected and 61.2% of the mares yielded at least one embryo.

In conformity with literature [44], holding of the oocytes prior to IVM did not significantly affect the maturation rate (*p* = 0.83), nor the cleavage (*p* = 0.78) or blastocyst rate (*p* = 0.78). The average AMH concentration of mares for which oocytes were subjected to holding prior to IVM was similar to the AMH level of mares whose oocytes were matured without prior holding.

The effect of the individual stallion on the blastocyst rate and on the number of blastocysts produced per OPU-ICSI session was not significant either (*p* = 0.07 for both).

A total of 64 OPU sessions were performed during Winter (from 21st of December to 20th of March), 37 during Autumn (from 21st of September to 20th of December) and 2 during Spring (from 21st of March to 20th of June). The season did not significantly affect the blastocyst rate or the number of blastocysts produced per OPU-ICSI session (*p* = 0.08 and *p* = 0.33, respectively). The effect of the season on the AMH concentration was not significant either (*p* = 0.76).

### 3.1. AMH and OPU-ICSI Outcome

**Figure 1:**
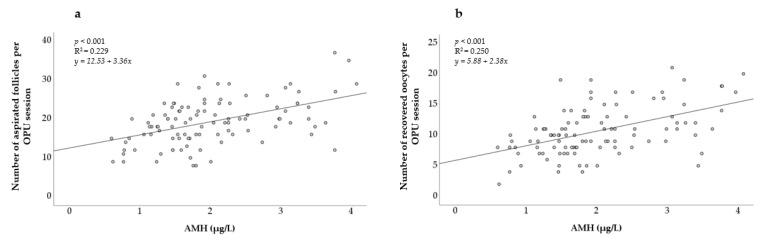
Association between AMH concentration and (**a**) number of aspirated follicles; (**b**) number of recovered oocytes. AMH = Anti-Müllerian hormone. R^2^ = coefficient of determination.

In Figure 1, a positive relationship between the AMH levels and both the number of aspirated follicles and the number of recovered oocytes can be observed. The linear regression was found to be highly significant (*p* < 0.001) for both models.

**Figure 2:**
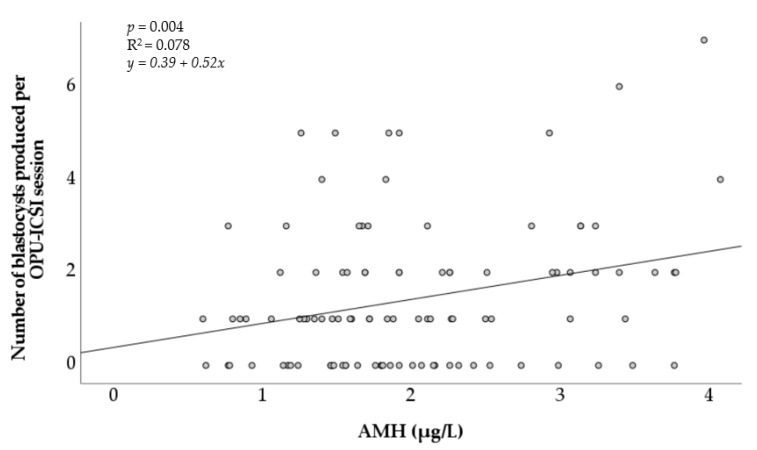
Association of the number of in vitro blastocysts produced after OPU-ICSI with the concentration of circulating anti-Müllerian hormone in individual mares. AMH = anti-Müllerian hormone. R^2^ = coefficient of determination.

The AMH concentration was also positively associated with the number of in vitro embryos produced per OPU-ICSI session (Figure 2). Although it was significant (*p* < 0.05), the positive linear relationship observed remained weak (R^2^ = 0.078).

To further explore the link between the circulating AMH concentration and the ICSI outcome, mares were divided according to serum AMH levels in the following three categories: Group 1 included the mares with AMH levels lower than 1.5 µg/L (*n* = 31 mares); Group 2 included the mares with AMH levels between 1.5 and 2.5 µg/L (*n* = 46 mares); Group 3 included the mares with AMH values above 2.5 µg/L (*n* = 26 mares). Mares whose AMH concentration was above 2.5 µg/L yielded significantly greater numbers of aspirated follicles, retrieved oocytes, mature oocytes, and cleaved embryos compared to mares with lower AMH concentrations (Table 1). Besides, mares from Group 3 produced significantly greater numbers of blastocysts per OPU-ICSI session compared to mares whose AMH concentration was below 1.5 µg/L. However, no significant differences were observed between groups for oocyte recovery rate, maturation rate, cleavage rate, or blastocyst rate.

**Figure 3:**
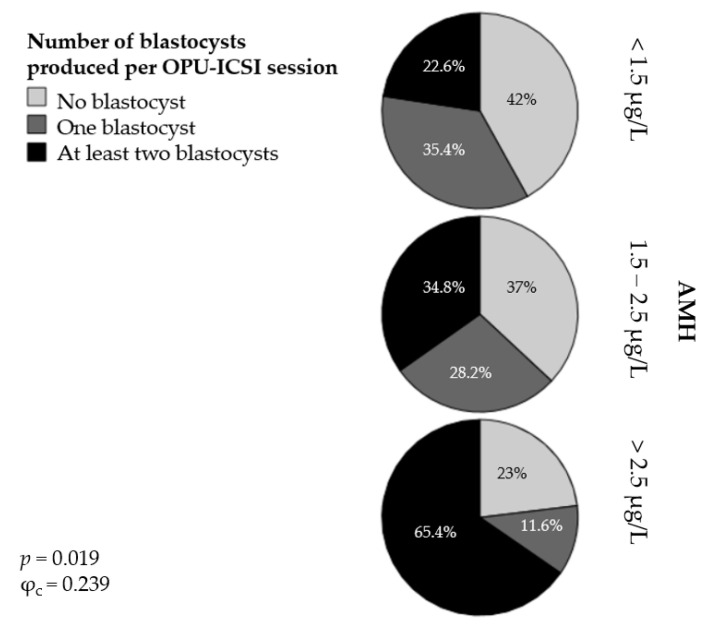
Percentages of mares producing no blastocysts, one blastocyst, or at least two blastocysts per OPU-ICSI session in three groups of mares with different serum AMH concentrations.

In addition, we classified the OPU-ICSI outcomes into three different categories according to the absolute number of blastocysts produced per OPU-ICSI session: no blastocysts, one blastocyst, and at least two blastocysts. The percentage of mares classified into the AMH groups and according to the absolute number of blastocysts produced per OPU-ICSI session is presented in Figure 3. In Group 1 ([AMH] < 1.5 µg/L), 42% of the mares produced no blastocysts following the OPU-ICSI procedure; 35.4% produced one blastocyst and 22.6% produced at least two blastocysts. In Group 2 ([AMH] = 1.5 to 2.5 µg/L), 37% of the mares produced no blastocysts, 28.2% produced one blastocyst, and 34.8% produced at least two blastocysts. Finally, in Group 3 ([AMH] > 2.5 µg/L), 23% of the mares failed to produce any blastocysts, 11.6% produced one blastocyst, and 65.4% produced at least two blastocysts. The association between the AMH groups and the categories of OPU-ICSI outcome was significant (*p* < 0.05) but weak (φ_c_ = 0.239).

### 3.2. AMH and Maternal Age

**Figure 4:**
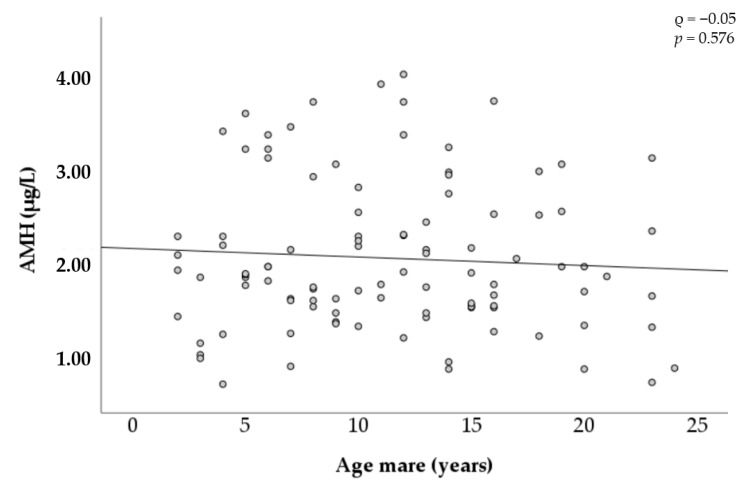
Relationship between age and AMH concentration in mares.

Figure 4 shows the changes in AMH concentration by the age of the mares. Considerable variation was observed between mares in all age categories. For instance, concentrations among mares between 2 and 4 years old ranged from 0.60 µg/L to 3.44 µg/L, and concentrations among mares between 20 and 24 years old ranged from 0.62 µg/L to 3.14 µg/L. No correlation was found to be significant among circulating AMH concentration and the age of the mares.

**Figure 5:**
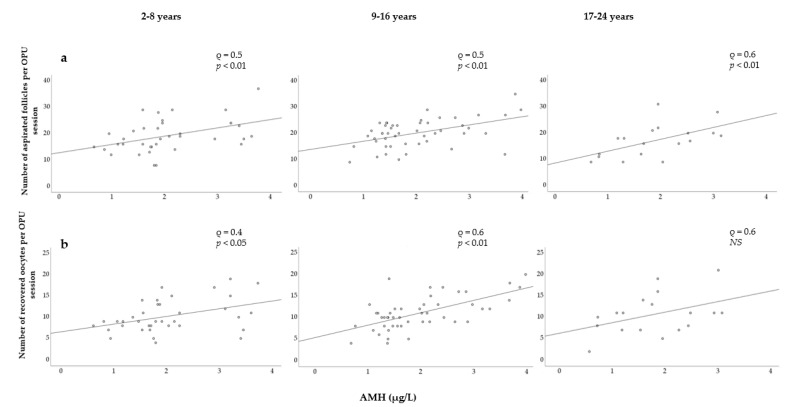
Relationship between serum AMH concentration and (**a**) the number of follicles aspirated; (**b**) the number of oocytes recovered in three age categories.

To evaluate possible effects of the mare’s age on other variables, mares were grouped into three categories: young (2–8 years), middle-aged (9–16 years), and old (17–24 years) (Figure 5). Strong and significant correlations were observed between the AMH concentration and the number of follicles within each group (Figure 5a). The correlation was slightly stronger in older mares, but no differences were found between the young and middle-aged mares. Strong and significant correlations were found between AMH levels and the number of oocytes retrieved in young and middle-aged mares (Figure 5b). The Spearman correlation coefficient was higher in middle-aged mares than in young mares, whereas correlation was found not to be significant in older mares.

No significant differences were observed between maternal age categories regarding the results after OPU-ICSI (Table 2).

### 3.3. AMH and OPU-ICSI Outcome within Individual Mares

**Figure 6:**
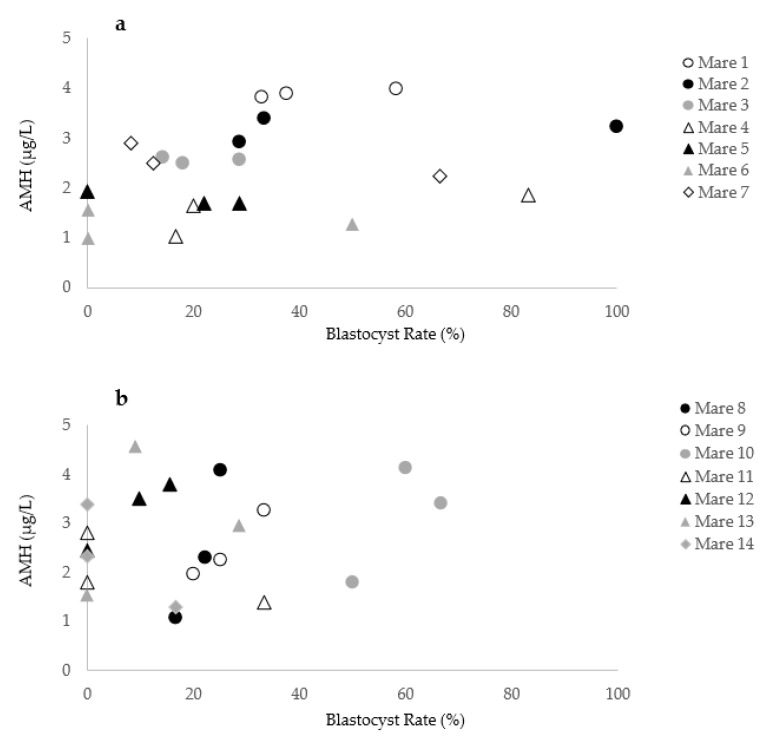
Variation in the blastocyst rate and AMH concentration in 14 individual mares during three OPU-ICSI cycles. (**a**) Mares 1 to 7 with similar AMH levels over different cycles.; (**b**) Mares 8 to 14 with a high variability in AMH levels over the consecutive cycles.

Fourteen of the 103 mares included in the study were presented in three consecutive OPU sessions. Blood samples for determination of serum AMH concentration were obtained at each procedure. In these 14 mares, over 42 OPU-ICSI sessions were performed using 13 different stallions. The rank of the OPU cycle (first vs. second vs. third) did not affect the AMH concentration (*p* = 0.6) or the blastocyst rate (*p* = 0.7). As descriptive observations, the individual variation in blastocyst rates and corresponding circulating AMH concentration over three consecutive OPU-ICSI sessions for 14 individual mares is shown in Figure 6. While the AMH levels of the mares 1, 2, 3, 4, 5, 6, and 7 appeared to be repeatable, a high variability of AMH levels over the OPU-ICSI cycles is observed for the mares 8, 9, 10, 11, 12, 13, and 14. According to the results of mare No. 9, AMH variation reflected the blastocyst rate (Cycle 1: AMH = 1.9 µg/L, blastocyst rate = 20%; Cycle 2: AMH = 3.2 µg/L, blastocyst rate = 33.3%; Cycle 3: AMH = 2.3 µg/L, blastocyst rate = 25%). However, while high variability in the AMH concentration of mare No. 8 was noticed (Cycle 1: AMH = 4.1 µg/L; Cycle 2: AMH = 1.1 µg/L; Cycle 3: AMH 2.3 µg/L), the blastocyst rate remained similar (Cycle 1: blastocyst rate = 25%; Cycle 2: blastocyst rate = 16.6%; Cycle 3: blastocyst rate = 22.2%). In mare No. 11, 33.3% of the oocytes injected became blastocysts while the AMH concentration was 1.4 µg/L. A blastocyst rate of 0% was observed in the same mare when the AMH levels rose to 2.8 µg/L.

## 4. Discussion

The overall OPU-ICSI results in this study are comparable to those reported elsewhere [1,45] with 61.2% of the mares producing at least one embryo and with an average of 1.4 (±0.2) blastocysts per session. Cuervo-Arango et al. [45] observed that a significantly greater number of blastocysts was obtained per session of OPU-ICSI (1.1 ± 1.4) than with embryo flush (0.64 ± 0.6). These competitive results lead to an increased popularity of OPU-ICSI for breeding valuable horses. Yet, the OPU-ICSI procedure remains complex and expensive. Therefore, establishment of a predictive model for OPU-ICSI outcome in mares would represent a useful tool to support client counselling and management of expectations. The success of equine in vitro embryo production is significantly influenced by many factors such as the identity of the donor stallions and mares, the antral follicle count, the number of oocytes recovered, and notably the clinician and laboratory expertise [5,6]. The oocyte developmental capacity appears to be less affected by the sperm donor than by the oocyte donor, leading to the hypothesis that a factor intrinsic to the mares could be used as a marker for success [6].

In this retrospective study, AMH was explored as a potential marker, due to its importance in folliculogenesis and its association with reproductive success in other mammals [13,14]. Indeed, mares whose AMH concentration was above 2.5 µg/L had more follicles aspirated, more oocytes recovered, and more in vitro embryos produced (2.1 ± 0.4 blastocysts). Overall, 77% of the mares from this category yielded at least one embryo after one session. The relationship between AMH and fertility has been investigated previously and results seem to be controversial. While thoroughbred mares with lower AMH values appeared to have a lower pregnancy rate after mating [41], Traversari et al. [46] observed no relationship between AMH concentration and pregnancy outcomes in warmblood mares. The current study indicates that the number of blastocysts produced per OPU session was significantly lower in mares whose AMH levels were below 1.5 µg/L. However, since no significant difference was observed in the maturation rate and blastocyst rate, one can assume that the greater number of blastocysts obtained in mares with higher levels of AMH is primarily due to a higher number of aspirated follicles and recovered oocytes.

In women, a positive correlation between serum anti-Müllerian hormone and live birth rate following IVF has been described [29,30]. It is worth noting that the influence of maternal age and weight hamper the use of AMH concentration as the only parameter to predict IVF outcome in women. Other researchers rejected the use of anti-Müllerian hormone concentrations as an independent predictor of the success of assisted reproductive therapy since acceptable pregnancy rates were obtained in patients with very low anti-Müllerian levels [47]. Moreover, the relationship between AMH concentration and oocyte quality is controversial in humans. Some studies observed a significant association between AMH concentration and embryo quality [48,49]. Interestingly, both very low (<1.66 ng/mL) and very high (>4.52 ng/mL) levels of AMH have been associated with a decrease in the quality of the oocytes affecting the implantation rate and pregnancy outcome [28]. However, the authors could not determine any basal AMH levels necessary to predict fertilization success or embryo quality. In a recent study, no significant difference was found regarding oocyte maturation or embryo quality in individuals with low AMH concentration (mean 0.67 ng/mL) when the number of retrieved oocytes was above seven [50]. Similarly, our data indicated no association between AMH concentration and oocyte or embryo quality since no significant differences were found between groups regarding maturation rate, cleavage rate and blastocyst rate. In cattle, Rico et al. [31] were able to determine a cut-off level of circulating AMH concentration, between 75 and 80 pg/mL, in order to discard low embryo producing cows after ovarian stimulation. Although identification of good or poor embryo donors might be possible, Vernunft et al. [51] dismissed the use of threshold values to assess the suitability of a cow for OPU since correlations between AMH level and success of a single OPU-IVF session were too low.

One should take into consideration that AMH levels, in women and domestic animals, actually predict the ability of the ovaries to respond to hyperstimulation with gonadotropin treatment rather than the spontaneous ability of the donor to produce an embryo. In women, maternal serum AMH concentrations display an important criterium to adapt the gonadotropin medication dose for IVF treatment, in combination with the serum concentration of follicle stimulating hormone (FSH) and the antral follicle count [52]. Interestingly, no correlation was found between serum AMH levels and pregnancy rates in subfertile women without medical therapy [53]. Likewise in sheep, plasma AMH concentrations provide a good parameter to predict the responsiveness to FSH treatment but were not representative of the follicular population in an ovary without gonadotropin stimulation [54]. To our knowledge, no study has been performed yet to determine the association between circulating AMH concentrations and superovulation treatment in mares. As a matter of fact, superovulation in mares remains a challenge [55]. Beneficial effects of eFSH treatment are only observed in the early vernal transition, and super-ovulatory treatment might even be related to impaired embryo quality [56,57]. Moreover, high individual variability in response to eFSH treatment predisposes mares to overstimulation of the ovary and can result in failure of ovulation or development of follicular cysts [55,58].

Measurement of circulating AMH as a marker of the ovarian function is often related to the maternal age [17]. Previous studies observed that AMH concentrations are significantly lower in older mares (16–27 years old), corresponding to a decline in the ovarian follicle pool [26,41]. However, it is worth mentioning that Traversari et al. [46] did not find such a relation and that Uliani et al. [42] observed a decrease in AMH concentrations only in mares older than 20 years. In agreement with previous observations, the correlation between the ovarian reserve and the amount of AMH in blood was shown to be stronger in older mares [26,46]. AMH levels were also positively correlated with the number of oocytes recovered in young and middle-aged mares. This association was not significant in old mares, which may be due to the smaller sample size of this group (18/103). A decrease in the number of aspirated follicles and recovered oocytes in a commercial OPU-ICSI program was also established in mares with increasing age, although the blastocyst rate was not affected [6,26]. The effect of the maternal age on AMH concentration, follicle and oocyte number was not observed in our data. Considering that the average number of follicles was similar in all age groups, due to selection of the mares prior to OPU, and that AMH concentration is primarily linked to follicular count, this lack of a decline could be expected. Similar to the aforementioned study, no effect of the maternal age on the reproductive efficiency of OPU-ICSI was observed in the present study, even though a trend towards lower blastocyst rate in older mares should be mentioned. It is worth emphasizing that the strict selection of our mares, based upon an optimal follicular count before the OPU-ICSI procedure, might mainly justify the lack of maternal age effect on circulating AMH concentrations, follicular count, and oocytes recovered.

A large variation in circulating AMH levels was observed between mares, ranging from 0.6 to 4.1 µg/L. This result is in agreement with previous studies where serum AMH concentrations ranged from 0.22 ng/mL to 2.94 ng/mL in normal cycling mares [35], from 0.07 to 3.56 ng/mL in pre-ovulatory mares [46], and from 0.26 ng/mL to 15.47 ng/mL in mares irrespective of the reproductive state [42]. Circulating AMH levels have been suggested to be related to the population of follicles measuring 6 to 20 mm [26]. Although all mares were selected to have at least 10 follicles between 5 and 25 mm, a high individual variability in AMH concentration was observed. Previous reports suggested an influence on the expression of AMH of the viability of the follicles that are lacking in atretic follicles [39]. Moreover, the reproductive stage may also affect the AMH concentration variability. Results from various studies regarding the consistency of AMH concentrations throughout the estrous cycle are inconclusive. Almeida et al. [35] found no significant difference in AMH concentration measured three times a week throughout one inter-ovulatory period in six normal cyclic mares, while Dal et al. [59] observed that AMH concentrations were significantly higher in estrus than in diestrus in 25 mares. As our study was designed retrospectively, from a clinical point view, and since AMH concentrations appear to be repeatable within individual normal cyclic mares [35,41], blood samples were obtained on the day of the OPU session, regardless of the day of the estrous cycle.

Moderate to strong correlations of AMH concentrations were found within and across breeding seasons for individual mares [41], and the in vitro embryo production capacity of donor mares was shown to be repeatable across OPU-ICSI procedures [45]. In addition, one recent abstract reported the usefulness of determination of AMH concentration as a predictor of oocyte recovery and in vitro production of equine embryo in warmblood mares [60]. Nonetheless, one caveat of the present study is the high variability between individual mares regarding the association between AMH concentration and blastocyst rate. The blastocyst rate is also affected by other factors, like semen quality [3]. Yet, the effect of the individual stallion on the OPU-ICSI outcome is less prominent than the variation between individual mares and no significant effect was observed in our study. Finally, even though a high AMH level is associated with higher embryo production, mares with low AMH values should still be considered suitable for commercial OPU-ICSI. In fact, in the current study, 58% of the mares with a low AMH concentration were able to produce at least one embryo. The high individual variation might limit the usefulness of serum AMH measurement above antral follicular count by ultrasonography to select mares with a high potential to produce equine in vitro embryos.

## 5. Conclusions

We conclude that a high AMH blood level at the moment of OPU is associated with a high number of oocytes recovered and a high number of embryos produced in a commercial OPU-ICSI program. However, the measurement of circulating AMH cannot be used as an independent predictor of the OPU-ICSI outcome. Even with a low AMH level, mares were able to produce at least one embryo. Selection of the mare by transrectal ultrasonography and assessment of the follicle count stand to be the main criteria of suitability to undergo a successful OPU-ICSI session.

## Figures and Tables

**Table 1:** Effect of serum AMH concentration on the OPU-ICSI outcome in mares.

AMH Group	Group 1 (<1.5 µg/L)	Group 2 (1.5–2.5 µg/L)	Group 3 (>2.5 µg/L)
Age	12.3 ± 1.1	10.8 ± 0.8	11.5 ± 1.0
No. Aspirated Follicles	16.5 ± 0.8 ^a^	19.2 ± 0.8 ^a^	22.9 ± 1.2 ^b^
No. Recovered Oocytes	8.6 ± 0.6 ^a^	10.5 ± 0.5 ^a^	13.5 ± 0.8 ^b^
Recovery Rate	53.3 ± 3.1	55.2 ± 1.6	58.1 ± 2.8
No. MII Oocytes	5.5 ± 0.5 ^a^	6.5 ± 0.3 ^a^	9.3 ± 0.7 ^b^
Maturation Rate (%)	66.4 ± 4.4	64.7 ± 2.8	68.9 ± 1.9
No. Cleaved	3.5 ±0.4 ^a^	4.2 ± 0.2 ^a^	6.2 ± 0.5 ^b^
Cleavage Rate (%)	67.1 ± 7.4	69.5 ± 5.0	66.8 ± 3.6
No. Blastocysts	1.1 ± 0.3 ^a^	1.2 ± 0.2 ^a, b^	2.1 ± 0.4 ^b^
Blastocyst Rate (%)	21.0 ± 5.2	20.5 ± 3.5	21.0 ± 3.4

AMH = anti-Müllerian hormone. OPU = ovum pick-up. ICSI = intracytoplasmic sperm injection. No = Number of. MII Oocytes = Oocytes in Metaphase II. Cleaved = Cleaved embryos on Day 2–3. Recovery Rate = No. Oocytes Recovered/No. Follicles Aspirated. Maturation Rate = No. MII Oocytes/No. Oocytes Recovered. Cleavage Rate = No. Cleaved embryos/No. MII Oocytes. Blastocyst Rate = No. Blastocysts/No. MII oocytes. Data are shown as mean value ± standard error of the mean (SEM). Within rows, different superscript letters (a,b) indicate significant differences (*p* < 0.05). The absence of superscripts indicates the lack of statistical differences between groups (*p* > 0.05).

**Table 2:** Effect of maternal age on OPU-ICSI outcome.

Age (years)	2–8	9–16	17–24
AMH (µg/L)	2.0 ± 0.1	2.1 ± 0.1	1.8 ± 0.2
No. Aspirated Follicles	19.2 ± 1.0	20.2 ± 0.8	17.1 ± 1.5
No. Recovered Oocytes	10.2 ± 0.6	11.0 ± 0.6	10.7 ± 0.4
Recovery Rate	53.7 ± 2.1	54.2 ± 1.9	61.8 ± 3.7
No. MII Oocytes	6.8 ± 0.5	7.1 ± 0.4	6.6 ± 0.7
Maturation Rate (%)	68.2 ± 3.1	65.5 ± 2.8	64.5 ± 5.1
No. Cleaved	4.4 ± 0.4	4.6 ± 0.3	4.4 ± 0.6
Cleavage Rate (%)	66.4 ± 3.8	70.4 ±5.9	65.0 ± 5.2
No. Blastocysts	1.5 ± 0.2	1.5 ± 0.2	1.6 ± 0.4
Blastocyst Rate (%)	20.8 ± 2.9	24.1 ± 4.1	12.6 ± 4.1

AMH: anti-Müllerian hormone. OPU: ovum pick-up. ICSI: intracytoplasmic sperm injection. No = Number of. MII Oocytes = Oocytes in Metaphase II. Cleaved = Cleaved embryos on Day 2–3. Recovery Rate = No. Oocytes Recovered/No. Follicles Aspirated. Maturation Rate = No. MII Oocytes/No. Oocytes Recovered. Cleavage Rate = No. Cleaved embryos/No. MII Oocytes. Blastocyst Rate = No. Blastocysts/No. MII oocytes. Data are shown as mean value ± standard error of the mean (SEM). The absence of subscripts indicates the lack of significant statistical differences (*p* > 0.05) between age related groups of mares.

## References

[1] Lazzari G., Colleoni S., Crotti G., Turini P., Fiorini G., Barandalla M., Landriscina L., Dolci G., Benedetti M., Duchi R. (2020). Laboratory production of equine embryos. J. Equine Vet. Sci..

[2] Hinrichs K. (2018). Assisted reproductive techniques in mares. Reprod. Domest. Anim..

[3] Stout T.A.E. (2020). Clinical application of in vitro embryo production in the horse. J. Equine Vet. Sci..

[4] Lazzari G., Crotti G., Turini P., Duchi R., Mari G., Zavaglia G., Barbacini S., Galli C. (2002). Equine embryos at the compacted morula and blastocyst stage can obtained by intracytoplasmic sperm injection (ICSI) of in vitro matured oocytes with frozen-thawed spermatozoa from semen of different fertilities. Theriogenology.

[5] Claes A.N.J., Galli C., Colleoni S., Necchi D., Lazzari G., Deelen C., Beitma M., Stout T. (2016). Factors influencing oocyte recovery and in-vitro production of equine embryos in a commercial OPU/ISCI program. J. Equine Vet. Sci..

[6] Cuervo-Arango J., Claes A.N., Stout T.A.E. (2019). Mare and stallion effects on blastocyst production in a commercial equine ovum pick-up-intracytoplasmic sperm injection program. Reprod. Fertil. Dev..

[7] Galli C., Colleoni S., Duchi R., Lazzari G. (2016). Male factors affecting the success of equine in vitro embryo production by ovum pickup-intracytoplasmic sperm injection in a clinical setting. J. Equine Vet. Sci..

[8] Josso N., Cate R.L., Picard J.Y., Vigier B., Di Clemente N., Wilson C., Imbeaud S., Pepinsky R.B., Guerrier D., Boussin L. (1993). Anti-müllerian hormone: The Jost factor. Recent Prog. Horm. Res..

[9] Durlinger A.L., Visser J.A., Themmen A.P. (2002). Regulation of ovarian function: The role of anti-Müllerian hormone. Reproduction.

[10] Josso N., Picard J.Y. (1986). Anti-Müllerian hormone. Physiol. Rev..

[11] Durlinger A.L., Kramer P., Karels B., de Jong F.H., Uilenbroek J.T., Grootegoed J.A., Themmen A.P. (1999). Control of primordial follicle recruitment by anti-Müllerian hormone in the mouse ovary. Endocrinology.

[12] Durlinger A.L., Gruijters M.J., Kramer P., Karels B., Kumar T.R., Matzuk M.M., Rose U.M., de Jong F.H., Iulenbroek J.T., Grootegoed J.A. (2001). Anti-Müllerian hormone attenuates the effects of FSH on follicle development in the mouse ovary. Endocrinoly.

[13] La Marca A., Sighinolfi G., Radi D., Argento C., Baraldi E., Carducci Artenisio A., Stabile G., Volpe A. (2010). Anti-Müllerian (AMH) as a predictive marker in assisted reproductive technology (ART). Hum. Reprod. Update.

[14] Umer S., Zhao S.J., Sammad A., Weldegebriall Sahlu B., Pang Y.W., Zhu H. (2019). AMH: Could it be used as a biomarker for fertility and superovulation in domestic animals?. Genes.

[15] Monniaux D., Drouilhet L., Rico C., Estienne A., Jarrier P., Touzé J.L., Sapa J., Phocas F., Dupont J., Dalbiès-Tran R. (2013). Regulation of anti-Müllerian hormone production in domestic animals. Reprod. Fertil. Dev..

[16] Fanchin R., Schonäuer L.M., Righini C., Guibourdenche J., Frydman R., Taieb J. (2003). Serum anti-Müllerian hormone is more strongly related to ovarian follicular status than serum inhibin B, estradiol, FSH and LH on day 3. Hum. Reprod..

[17] Hansen K.R., Hodenett G.M., Knowlton N., Craig L.B. (2011). Correlation of ovarian reserve tests with histologically determined primordial follicle number. Fertil. Steril..

[18] Kevenaar M.E., Meerasahib M.F., Kramer P., van de Lang-Born B.M.N., de Jong F.H., Groome N.P., Themmen A.P.N. (2006). Serum anti-Müllerian hormone level reflects the size of the primordial follicle pool in mice. Endocrinology.

[19] Batista E.O.S., Macedo G.G., Sala R.V., Ortolan M.D.D.V., Filho M.F.S., Del Valle T.A., Jesus E.F., Lopes R.N.V.R., Rennó F.P., Baruselli P.S. (2014). Plasma antimullerian hormone as a predictor of ovarian antral follicular population in Bos indicus (Nelore) and Bos Taurus (Holstein) heifers. Reprod. Domest. Anim..

[20] Nagashima J.B., Hansen B.S., Songsasen N., Travis A.J., Place N.J. (2016). Anti-Müllerian hormone in the domestic dog during the anestrus to oestrus transition. Reprod. Domest. Anim..

[21] Vernunft A., Schneider F., Tuchscherer F., Becker F., Kanitz W. (2011). Anti-Müllerian hormone (AMH) can help to predict follicular growth in mare. Pferdeheilkunde.

[22] Claes A.N.J., Ball B.A., Troedsson M.H.T., Curry JR T.E., Squires E.L., Scoggin K.E. (2015). Molecular changes in the equine follicles in relation to variations in antral follicle count and anti-Müllerian hormone concentrations. Equine Vet. J..

[23] Jayaprakasan K., Deb S., Batcha M., Hopkisson J., Johnson I., Campbell B., Raine-Fenning N. (2010). The cohort of antral follicles measuring 2–6 mm reflects the quantitative status of ovarian reserve as assessed by serum levels of anti-Müllerian hormone and response to controlled ovarian stimulation. Fertil. Steril..

[24] Rico C., Fabre S., Médigue C., di Clemente N., Clément F., Bontoux M., Touzé J.L., Dupont M., Briant E., Rémy B. (2009). Anti-Müllerian hormone is an endocrine marker of ovarian gonadotropin-responsive follicles and can help to predict superovulatory response in cow. Biol. Reprod..

[25] Monniaux D., Baril G., Laine A.L., Jarrier P., Poulin N., Cognié J., Fabre S. (2011). Anti-Müllerian hormone as a predictive endocrine marker for embryo production in the goat. Reproduction.

[26] Claes A.N.J., Ball B.A., Scoggin K.E., Esteller-Vico A., Kalmar J.J., Conley A.J., Squires E.L., Troedsson M.H.T. (2015). The interrelationship between anti-Müllerian hormone, ovarian follicular populations and age in mares. Equine Vet. J..

[27] Dewailly D., Yding Andersen C., Balen A., Broekmans F., Dilaver N., Fanchin R., Griesinger G., Kelsey T.W., La Marca A., Lambalk C. (2014). The physiology and clinical utility of anti-Müllerian hormone in women. Hum. Reprod. Update.

[28] Ebner T., Sommergruber M., Moser M., Shebl O., Schreier-Lechner E., Tews G. (2006). Basal level of anti-Müllerian hormone is associated with oocyte quality in stimulated cycled. Hum. Reprod..

[29] Brodin T., Hadziosmanovic N., Berglund L., Olovsson M., Holte J. (2015). Comparing four ovarian reserve markers—Associations with ovarian response and live births after assisted reproduction. Acta Obstet. Gynecol. Scand..

[30] Hu K.L., Liu F.T., Xu H., Li R., Qiao J. (2020). Association of serum anti-Müllerian hormone and other factors with cumulative live birth rate following IVF. RBMO.

[31] Rico C., Drouilhet L., Salvetti P., Dalbiès-Tran R., Jarrier P., Touzé J.L., Pillet E., Ponsart C., Fabre S., Monniaux D. (2012). Determination of anti-Müllerian hormone concentrations in blood as a tool to select Holstein donor cows for embryo production: From the laboratory to the farm. Reprod. Fertil. Dev..

[32] Fushimi Y., Okawa H., Monniaux D., Takagi M. (2020). Efficacy of a single blood anti-Müllerian hormone (AMH) concentration measurement for the selection of Japanese Black heifer embryo donors in herd breeding program. J. Reprod. Dev..

[33] Steel A., Athorn R.Z., Grupen C.G. (2018). Anti-Müllerian hormone and oestradiol as markers of future reproductive success in juveniles gilts. Anim. Reprod. Sci..

[34] Am-in N., Suwimonteerabutr J., Kirkwood R.N. (2020). Serum anti-Müllerian hormone and estradiol concentrations in gilts and their age at puberty. Animals.

[35] Almeida J., Ball B.A., Conley A.J., Place N.J., Liu I.K.M., Scholtz E.L., Mathewson L., Stanley S.D., Moeller B.C. (2011). Biological and clinical significance of Anti-Müllerian hormone determination in blood serum of the mare. Theriogenology.

[36] Claes A.N.J., Ball B.A. (2016). Biological functions and clinical applications of anti-Müllerian hormone in stallions and mares. Vet. Clin. Equine.

[37] Claes A.N.J., Ball B.A., Corbin C.J., Conley A.J. (2014). Anti-Müllerian hormone as a diagnostic marker for equine cryptorchidism in three cases with equivocal testosterone concentrations. J. Equine Vet. Sci..

[38] Ball B.A., Conley A.J., MacLaughlin D.T., Grundy S.A., Sabeur K., Liu I.K.M. (2008). Expression of anti-Müllerian hormone (AMH) in equine granulosa-cell tumor and in normal equine ovaries. Theriogenology.

[39] Scarlet D., Wulf M., Kuhl J., Köhne M., Ille N., Conley A.J., Aurich C. (2018). Anti-Müllerian hormone profiling in prepubertal horses and its relationship with gonadal function. Theriogenology.

[40] Joonè C.J., Schulman M.L., Fosgate G.T., Claes A.N.J., Gupta S.K., Botha A.E., Human A., Bertschinger H.J. (2018). Serum anti-Müllerian hormone dynamics in mares following immunocontraception with anti-zona pellucida or -GnRH vaccines. Theriogenology.

[41] Ball B.A., El-Sheikh Ali H., Scoggin K.E., Riddle W.T., Schnobrich M., Bradecamp E., Agnew M., Squires E.L., Troedsson M.H.T. (2019). Relation between anti-Müllerian hormone and fertility in the mare. Theriogenology.

[42] Uliani R.C., Conley A.J., Corbin C.J., Friso A.M., Maciel L.F.S., Alvarenga M.A. (2019). Anti-Müllerian hormone and ovarian aging in mares. J. Endocrinol..

[43] Ortiz-Escribano N., Bogado Pascottini O., Woelders H., Vandenberghe L., De Schauwer C., Govaere J., Van den Abbeel E., Vullers T., Ververs C., Roels K. (2018). An improved vitrification protocol for equine immature oocytes resulting in a first live foal. Equine Vet. J..

[44] Choi Y.H., Love L.B., Varner D.D., Hinrichs K. (2006). Holding immature equine oocytes in the absence of meiotic inhibitors: Effect on germinal vesicle chromatin and blastocyst development after intracytoplasmic sperm injection. Theriogenology.

[45] Cuervo-Arango J., Claes A.N., Stout T.A. (2019). A retrospective comparison of the efficiency of different assisted reproductive techniques in the horse, emphasizing the impact of maternal age. Theriogenology.

[46] Traversari J., Aepli H., Knutti B., Lüttgenau J., Bruckmaier R., Bollwein H. (2019). Relationships between antral follicle count, blood serum concentration of anti-Müllerian hormone and fertility in mares. Schweiz. Arch. Tierheilkd..

[47] Pacheco A., Cruz M., Iglesias C., García-Velasco J.A. (2018). Very low anti-müllerian hormone concentrations are not an independent predictor of embryo quality and pregnancy rate. Reprod. Biomed. Online.

[48] Melado Vidales L., Fernández-Nistal A., Martínez Fernández V., Verdú Merino V., Bruna Catalán I., Bajo Arenas J.M. (2017). Anti-Müllerian hormone levels to predict oocyte maturity and embryo quality during controlled ovarian hyperstimulation. Minerva Ginecol..

[49] Scheffer J.B., de Carvalho R.F., Aguiar A.P.S., Machado I.J.M., France J.B., Lozano D.M., Fanchin R. (2021). Which ovarian reserve marker relates to embryo quality on day 3 and blastocyst; age, AFC, AMH?. JBRA Assist. Reprod..

[50] Wu W., Wang X., Li Y., Zhang Y. (2020). Analysis of the women with the AMH concentrations below the limit of reference range but with the ideal number of retrieved oocytes. Arch. Gynecol. Obstet..

[51] Vernunft A., Schwerhoff M., Viergutz T., Dierderich M., Kuwer A. (2015). Anti-Müllerian hormone levels in plasma of Holstein-Friesian heifers as a predictive parameter for ovum pick-up and embryo production outcomes. J. Reprod. Dev..

[52] Sadruddin S., Barnett B., Ku L., Havemann D., Mucowski S., Herrington R., Burggren W. (2020). Maternal serum concentration of anti-Müllerian hormone is a better predictor than basal follicle stimulating hormone of successful blastocysts development during IVF treatment. PLoS ONE.

[53] Casadei L., Manicuti C., Puca F., Madrigale A., Emidi E., Piccione E. (2013). Can anti-Müllerian hormone be predictive of sponteneus onset of pregnancy in women with unexplained infertility?. J. Obstet. Gynaecol..

[54] Lahoz B., Alabart J.L., Cocero M.J., Monniaux D., Echegoyen E., Sánchez P., Folch J. (2014). Anti-Müllerian hormone concentration in sheep and its dependence of age an independence of *BMP15* genotype: An endocrine predictor to select the best donors for embryo technologies. Theriogenology.

[55] Squires E.L., McCue P.M. (2007). Superovulation in mares. Anim. Reprod. Sci..

[56] Raz T., Hunter B., Carley S., Card C. (2009). Reproductive performance of donor mares subsequent to eFSH treatment in early vernal transition: Comparison between the first, second, and mid-season estrous cycles of the breedinf season. Anim. Reprod. Sci..

[57] Meyers-Brown G.A., McCue P.M., Troedsson M.H.T., Klein C., Zent W., Ferris R.A., Lindholm A.R.G., Scofield D.B., Claes A.N., Morganti M. (2013). Induction of ovulation in seasonally anestrous mares under ambient lights using recombinant equine FSH (reFSH). Theriogenology.

[58] Raz T., Card C. (2009). Efficiency of superovulation and in vivo embryo production in eFSH-treated donor mares after estrus synchronization with progesterone and estradiol-17β. Theriogenology.

[59] Dal G.E., Kasikci G. (2020). Serum anti-Müllerian hormone levels during estrus and diestrus in mares. Med. Weter.

[60] Claes A.N.J., Cuervo-Arango J., Derks S., Stout T.A.E. (2020). The usefulness of anti-Müllerian hormone in predicting oocyte recovery and in vitro production of equine embryos. J. Equine Vet. Sci..

